# Antimicrobial Activity of Peptides Derived from Olive Flounder Lipopolysaccharide Binding Protein/Bactericidal Permeability-Increasing Protein (LBP/BPI)

**DOI:** 10.3390/md12105240

**Published:** 2014-10-17

**Authors:** Bo-Hye Nam, Ji-Young Moon, Eun-Hee Park, Young-Ok Kim, Dong-Gyun Kim, Hee Jeong Kong, Woo-Jin Kim, Young Ju Jee, Cheul Min An, Nam Gyu Park, Jung-Kil Seo

**Affiliations:** 1 Biotechnology Research Division, National Fisheries Research and Development Institute, Haean-ro 216, Gijang-eup, Gijang-gun, Busan 619-705, Korea; E-Mails: moonjy@nfrdi.go.kr (J.-Y.M.); 549@nfrdi.go.kr (E.-H.P.); yokim@nfrdi.go.kr (Y.-O.K.); combikola@nfrdi.go.kr (D.-G.K.); konghj@nfrdi.go.kr (H.J.K.); wjkim@nfrdi.go.kr (W.-J.K.); philaqua@nfrdi.go.kr (Y.J.J.); ancm@nfrdi.go.kr (C.M.A.); 2 Department of Biotechnology, Pukyong National University, Busan 608-737, Korea; E-Mail: ngpark@pknu.ac.kr; 3 Department of Food Science and Biotechnology, Kunsan National University, Korea

**Keywords:** antimicrobial peptide, LPS-binding protein, bactericidal permeability-increasing protein, analogs, antimicrobial mechanism

## Abstract

We describe the antimicrobial function of peptides derived from the *C*-terminus of the olive flounder LBP BPI precursor protein. The investigated peptides, namely, ofLBP1N, ofLBP2A, ofLBP4N, ofLBP5A, and ofLBP6A, formed α-helical structures, showing significant antimicrobial activity against several Gram-negative bacteria, Gram-positive bacteria, and the yeast *Candida albicans*, but very limited hemolytic activities. The biological activities of these five analogs were evaluated against biomembranes or artificial membranes for the development of candidate therapeutic agents. Gel retardation studies revealed that peptides bound to DNA and inhibited migration on an agarose gel. In addition, we demonstrated that ofLBP6A inhibited polymerase chain reaction. These results suggested that the ofLBP-derived peptide bactericidal mechanism may be related to the interaction with intracellular components such as DNA or polymerase.

## 1. Introduction

Antimicrobial peptides (AMPs) may be applicable as antibiotic surrogates due to their robust killing activity against a broad spectrum of bacterial species. AMPs are a family of host-defense molecules involved in innate immunity, most of which are gene-encoded and produced by living organisms of all types from prokaryotes to mammals. AMPs are considered a promising drug candidate due to their broad range of activity, low toxicity, and decreased resistance development by target cells [1]. Methods to identify AMP sequences from genomic/transcriptome databases have been used for the identification of novel AMPs. In addition, novel AMPs have been designed based on the amino acid sequence of proteins with antibacterial or bactericidal activity [2,3].

Lipopolysaccharide binding protein (LBP), an acute phase protein produced during infections with Gram-negative bacteria, binds with high affinity to bacterial lipopolysaccharides (LPS), specifically to the lipid A portion [4]. The crystal structure of bactericidal permeability-increasing protein (BPI), which is an LPS-binding protein, is similar to LBP [5]. In humans, LBP and BPI are structurally similar with 45% sequence identity [6]. Although these two proteins have similar structures, the biological roles of LBP and BPI are different. LBP is a plasma protein that enhances the inflammatory response to LPS [4], whereas BPI is found in the lysosomal granules of polymorphonuclear neutrophils; BPI is bactericidal and neutralizes the toxic effects of LPS [7]. The structural and functional aspects of LBP and BPI have been well-studied in mammals. In non-mammals, including lower vertebrates and invertebrates, LBP or BPI homologs have not been classified as either a LBP or a BPI because the functional properties of these proteins have not been characterized. LBP/BPI-related genes have been identified in several fish such as rainbow trout (*Onchorhynchus mykiss*) [8], carp (*Cyprinus carpio*) [9], Atlantic cod (*Gadus morhua*) [10], and olive flounder (*Paralichthys olivaceus*) [11].

LBP and BPI consist of *N*- and *C*-terminal domains, which can be cleaved into two nearly equally sized fragments of ~200 amino acids and share a similar two layer α/β structure, but show little sequence identity to each other. The *N*-terminal domain in LBP binds to LPS, whereas the *C*-terminal domain is required for the delivery of LPS to CD14, an important component in the LPS-signaling cascade [12]. The antibacterial and LPS neutralizing activities of BPI are fully expressed by its *N*-terminal domain. A recombinant 23-kDa *N*-terminal fragment of BPI has equivalent or greater activity than the holoprotein in bactericidal and LPS binding assays [13,14]. Additionally, three cleavage fragments and a synthetic peptide, which were designed to overlap with the 23-kDa *N*-terminal fragment of BPI, were analyzed for biological activities and revealed that only one synthetic peptide (amino acids 85–99) was bactericidal [15]. However, the function of the *C*-terminal portion remains unclear [16].

In the present study, we designed a series of peptides based on the amino acid sequence of the *C*-terminal domain of the olive flounder LBP/BPI homolog to develop a novel antimicrobial peptide. Antimicrobial activities of peptides against Gram-positive and Gram-negative bacterial strains, as well as hemolytic activities, were examined. Furthermore, we investigated the action mechanism of peptides by measuring the permeabilization of biomembranes and analyzing fluorescent dye leakage from lipid vesicles mimicking bacterial membranes. In addition, DNA gel electrophoresis and polymerase chain reaction (PCR) were used to determine whether the peptides interacted with bacterial intracellular components. The overall objective of this report was to facilitate the development of novel peptides for treating microbe infections.

## 2. Results and Discussion

### 2.1. Peptide Design and Synthesis

To design a series of ofLBP analogs, the secondary structure of ofLBP (GenBank Accession No. EJ667947) was predicted using the GOR method [17]. Five α-helical regions were predicted at each *N*-terminal and *C*-terminal domain of ofLBP, respectively, and their antimicrobial activity was investigated. The p*I* value, net positive charge, and Boman Index were predicted (Table 1). Factors such as hydrophobicity, net charge, and protein-binding potential (Boman Index) can have an influence on peptide activity. The Boman Index is an estimate of the potential of peptides to bind to other proteins, such as different receptors, and is defined as the sum of the free energies of the amino acid residue side chains divided by the total number of amino acid residues [18]. Among all peptides, a lower index value (≤1) suggests that the peptide has more antimicrobial activity without many side effects, whereas a higher index value (2.50–3.00) indicates that the peptide is multifunctional with hormone like activities [18]. Five parental peptides located at the *C*-terminal domain showed highly basic p*I* values (>10), positive net charge (+1 to +4), and lower Boman Index (≤1), while α-helical peptides in the N-terminal domain showed a comparatively lower net charge (−1 to +2) and higher Boman Index (0.33–1.78; Table 1).

**Figure 1:**
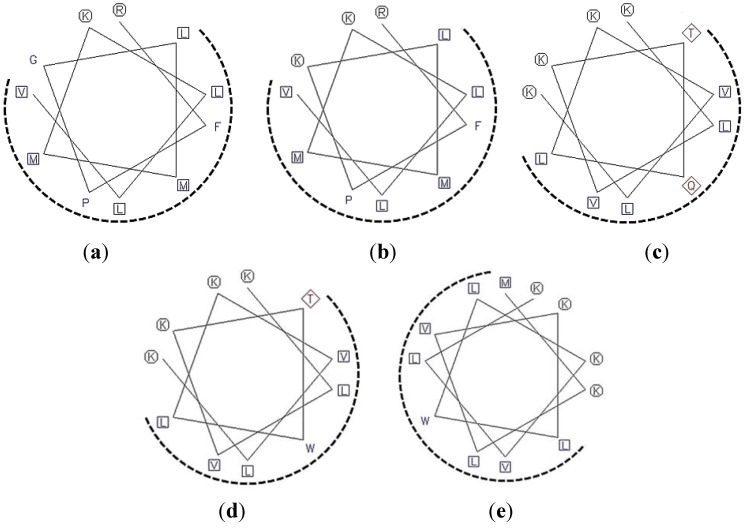
A Schiffer–Edmundson helical wheel representation of the analog peptides ofLBP1N (**a**); ofLBP2A (**b**); ofLBP4N (**c**); ofLBP5A (**d**); and ofLBP6A (**e**). The hydrophobic and hydrophilic residues are shown in a rectangular box and a circle, respectively. The hydrophobic face is indicated as a dashed arc.

Schiffer–Edmundson helical wheel projections were used to predict hydrophobic and hydrophilic regions in the secondary structure of two selected regions (Figure 1). This plot showed that ofLBP analogs adopted an amphipathic structure in which hydrophobic and hydrophilic residues containing basic residues were positioned on opposite sides. The distribution of hydrophobic and charged residues strongly affects the antimicrobial activity of AMPs such as pleurocidin and piscidins [19].

To design a series of ofLBP analogs, three different concepts were used: substitution of amino acids, addition of amino acids, or amidation at the *C*-terminus. To increase helicity and amphipathicity, Gly or Gln residues in the selected regions were substituted for a charged (Lys) or aromatic (Trp) residue, or a Met residue was added to the *N*-terminus of each analog (Table 1). In addition, to increase resistance to carboxypeptidase-type digestion *in vivo* and increase the positive charged state for the interaction of analogs with bacterial membranes, the *C*-terminus was amidated. Figure 1 shows helical-wheel representations of analog peptides with the positions of the amino acid substitutions or additions. Lys residues were introduced to verify positive charge effects on antimicrobial activity [20]. A Trp residue was also introduced to examine its ability to increase antimicrobial activity and reduce hemolytic activity [20]. The presence of several positive charges (cationic) combined with an amphipathic structure suggested that ofLBP analogs target the bacterial membrane. The antimicrobial activities of the analogs were tested against Gram-positive and Gram-negative bacteria, as well as *C. albicans*. All peptides were obtained using a solid-phase synthesis method with a good yield (>95%). Table 1 shows all peptide sequences used in this study.

**Table 1:** Summary of design strategy and overall characteristics of ofLBP analogs.

Domain	Amino Acid Sequence in α-Helix Predicted	Position/AA Length	p*I* Value	Net Charge	Boman Index * (kcal/mol)	Hydrophobic Ratio * (%)	Hydrophobicity* (kcal/mol)	Modification
*N*-terminal domain	LEYGRQLGMASIQQKLK	32–48/17	10.21	+2	1.6	35	3.71	
	VEYSLSHMQIVKL	67–79/13	7.76	0	0.33	46	1.56	
	WLYNLFKNFIDKALRNALQKQ	170–190/21	10.44	+3	1.66	47	0.17	
	VNELNPHLKTLNVLAKVDQYAE	199–220/22	5.37	−1	1.4	40	7.22	
	SIDLN	235–239/5	3.0	−1	1.78	40	0.91	
*C*-terminal domain	AYSVNSAAFVYNK	276–288/13	9.65	+1	0.72	46	−0.27	
	EISKRFPGLMMKLLVQ	320–335/16	10.59	+2	0.58	50	2.47	
	RFPGLMMKLLV ^a^	324–334/11	11.51	+2	−0.63	63	−0.94	
	RFPKLMMKLLV ^b^	324–334/11	11.67	+3	−0.04	63	0.04	Gly→Lys
	MRVAGAVSLN	389–398/10	10.9	+1	0.5	60	1.06	
	FKVRSLDNILQMVLKVVVI	412–430/19	10.53	+1	0.52	63	1.84	
	KMKLVKTQLKVLKDYMLI	448–465/18	10.45	+4	0.4	50	3.09	
	KLVKTQLKVLK^c^	450–460/11	10.98	+4	0.67	45	3.14	
	KLVKTWLKVLK^d^	450–460/11	10.98	+4	−0.03	54	0.71	Gln→Trp
	MKLVKTWLKVLK^e^	449–460/12	10.99	+4	−0.22	58	0.48	Gln→Trp

* Boman index, hydrophobic ratio and hydrophobicity were calculated by the predictive tool available at Antimicrobial Peptide Database v2.34 (APD2); ^a^ ofLBP1N; ^b^ ofLBP2A; ^c^ ofLBP4N; ^d^ ofLBP5A; ^e^ ofLBP6A. The parent sequences for the design of analogs were underlined.

### 2.2. Antimicrobial and Hemolytic Activities

The antimicrobial activity of analogs was determined by measuring minimal effective concentrations (MECs) against several bacterial species and one yeast (*C. albicans*) using URDA (Table 2). The antimicrobial activities of four analogs (ofLBP 1N, ofLBP 2A, ofLBP 5A, and ofLBP 6A) were similar or slightly weaker than piscidin 1, which was used as a positive control. The four analogs showed strong antimicrobial activity against Gram-positive bacteria including two *B. subtilis* strains, *M. luteus*, and *S. aureus* (MECs 0.7–8.0 μg/mL), and Gram-negative bacteria including three *E. coli* strains, *P. aeruginosa*, and *S. enterica* (MECs 0.5–8.0 μg/mL). Notably, four analogs exhibited potent activity against some fish pathogens including *A. hydrophila*, *S. iniae*, and two *V. parahemolyticus* strains (MECs 2.8–5.3 μg/mL), but showed less activity against *E. tarda* (MECs ≥ 62.5 μg/mL) and *V. alginolyticus* (MECs ≥ 20.0 μg/mL). They also showed potent activity against *C. albicans* (MECs 2.7–5.9 μg/mL). ofLBP 4N showed much less activity than the four analogs against all tested strains, excluding *E. coli* KCTC1116 and *V. parahemolyticus* KCCM41664. These results indicated that the four analogs had broad spectrum antimicrobial activity.

**Table 2:** MEC for antimicrobial activities of ofLBP-derived synthetic peptides.

Microbes	Gram	Minimal Effectives Concentration (μg/mL)					
		ofLBP 1N	ofLBP 2A	ofLBP 4N	ofLBP 5A	ofLBP 6A	Piscidin 1
*B. subtilis* KCTC1021	+	1.4	0.8	62.5	0.7	1.4	2.3
*B. subtilis* RM125	+	1.0	1.2	250.0	4.2	1.8	4.5
*M. luteus* ATCC9341	+	5.3	5.7	250.0	8.0	2.2	2.3
*S. aureus* RM4220	+	5.6	5.0	62.5	3.7	2.7	2.2
*E. coli* D31	−	1.3	0.5	62.5	0.9	2.6	2.0
*E. coli* KCTC1116	−	1.7	1.2	1.5	1.6	3.8	7.0
*E. coli* ML35p	−	0.8	0.6	250.0	1.6	1.8	2.3
*P. aeruginosa* KCTC2004	−	6.4	6.6	>250.0	8.0	4.6	8.0
*S. enterica* KCTC2514	−	1.2	1.2	250.0	3.5	2.6	7.0
**Fish pathogen**							
*A. hydrophila* KCTC2358	−	4.8	3.6	>250.0	5.0	2.8	10.0
*E. tarda* H-4	−	125.0	62.5	>250.0	250.0	62.5	125.0
*S. iniae* FP5229	+	5.3	3.0	>250.0	2.7	1.2	6.5
*V. parahemolyticus* KCCM41664	−	2.2	1.7	1.3	1.2	0.9	1.8
*C. albicans* KCTC7965	Yeast	5.9	5.6	125.0	4.0	2.7	>62.5

**Figure 2:**
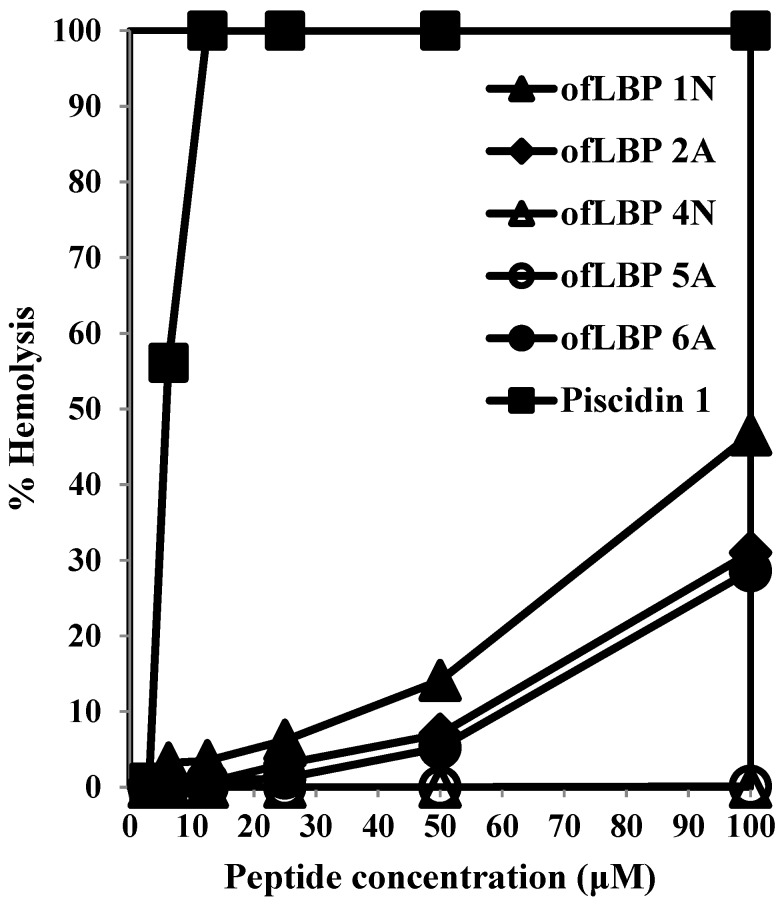
Hemolytic activities of ofLBP analog peptides and piscidin 1 against human erythrocytes.

To examine cytotoxicity, the hemolytic activity of the analogs and piscidin 1 was determined using human RBCs (Figure 2). ofLBP 4N and ofLBP 5A did not cause hemolysis against RBCs up to concentrations of 100 μg/mL. However, ofLBP 1N, 2A, and 6A did not show significant hemolytic activity at 50 μg/mL concentrations, but exhibited some hemolytic activity (~28%–50%) from concentrations of 50–100 μg/mL. In contrast, piscidin 1 caused strong hemolysis even at concentrations of 12.5 μg/mL. These results suggested that ofLBP 4N and ofLBP 5A were not cytotoxic, but ofLBP 1N, ofLBP 2A, and ofLBP 6A have some cytotoxicity (less than piscidin 1). In addition, ofLBP analogs have low cytotoxicity and may be applicable as alternative therapeutic agents for humans or can be used as an alternative to conventional antibiotics for the treatment or control of fish disease.

### 2.3. Killing Kinetic Study

**Figure 3:**
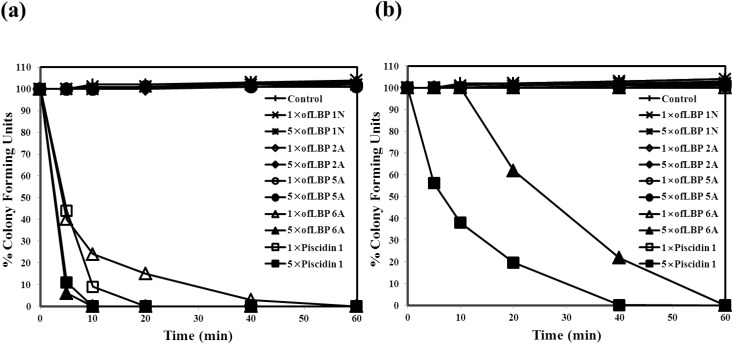
Bacterial killing kinetics of ofLBP analog peptides and piscidin 1 against *Bacillus subtilis* KCTC1021 (**a**) and *Escherichia coli* ML35p (**b**). Bacteria were incubated in the presence of 1× or 5× MEC of ofLBP analog peptides or piscidin 1. Control samples did not contain a peptide.

To evaluate the inhibition mode of analogs, a killing kinetic study was performed against *B. subtilis* KCTC1021 and *E. coli* ML35p at their MECs and at five times their MECs (Figure 3). ofLBP 4N was not studied due to lack of activity against the two bacterial species used in this report. Analogs (excluding ofLBP 6A) did not kill the two bacterial strains at all tested concentrations. However, ofLBP 6A and piscidin 1 rapidly killed *B. subtilis* KCTC1021 within 10 min at the 5× MEC and within 40 min at the 1× MEC. To confirm the target site of antimicrobial activity, a killing kinetic study was performed against *E. coli* ML35p at its MEC and at five times its MEC. ofLBP 6A and piscidin 1 did not kill *E. coil* ML35p at the 1× MEC. However, ofLBP 6A and piscidin 1 killed this strain within 60 and 40 min, respectively, at the 5× MEC. These killing kinetic studies indicated that the inhibition mode of ofLBP 6A on the two bacteria is bactericidal rather than bacteriostatic, but that other analogs function through bacteriostatic processes. Similar to ofLBP 6A, piscidin 1 acted through a bactericidal process.

### 2.4. Membrane Permeabilization Ability

**Figure 4:**
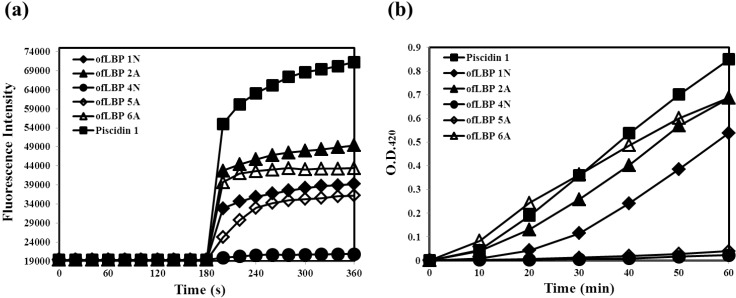
Outer and cytoplasmic membrane permeabilization of *Escherichia coli* ML35p by ofLBP analog peptides and piscidin 1. (**a**) Permeabilization of the outer membrane was monitored as an increase in NPN fluorescence intensity in the presence of peptides (40 μg/mL); (**b**) Cytoplasmic membrane permeabilization was monitored as an increase in fluorescence intensity by the hydrolysis of the impermeable, chromogenic substrate ONPG in the presence of peptides (40 μg/mL).

To determine whether ofLBP analogs permeabilize bacterial membranes, the membrane permeabilization ability of ofLBP analogs against *E. coli* ML35p was measured (Figure 4). Permeabilization of the outer membrane was monitored using the NPN uptake assay [21]. NPN fluoresces weakly in an aqueous environment but strongly in the hydrophobic interior of cell membranes. Upon destabilization of the cellular membrane by AMPs, the dye enters the damaged membrane where it emits stronger fluorescence. The fluorescence from *E. coli* ML35p was monitored after incubation with NPN and the peptides. ofLBP analogs showed significant outer membrane permeabilization ability: 2A > 6A > 1N > 5A. Piscidin 1 showed strong permeabilization ability; the permeability was stronger than that of the ofLBP analogs (Figure 4). The permeabilization of the *E. coli* ML35p inner cytoplasmic membrane was also measured using the β-galactosidase assay [8]. *E. coli* ML35p was incubated with the peptides and ONPG. The hydrolysis of ONPG (a chromogenic substrate) by cytoplasmic β-galactosidase was measured spectrophotometrically at 420 nm. Similar to the results of the NPN uptake assay, ofLBP 6A, 2A, and 1N showed strong permeabilization ability, whereas ofLBP 5A and 4N showed no permeabilization ability. Piscidin 1 also displayed strong cytoplasmic membrane permeabilizing activity. These results suggested that ofLBP analogs can permeabilize bacterial membranes more efficiently than piscidin 1, which is known to strongly permeabilize the bacterial membrane [22]. Notably, ofLBP 5A did not show inner membrane permeability although it did exhibit outer membrane permeabilization ability, indicating that this peptide may not directly affect the inner bacterial membrane. However, ofLBP 4N showed no inner or outer membrane permeability, which is in agreement with the antimicrobial activity result. These phenomena differ from the results obtained using the killing kinetic study (Figure 3), which may be related to the concentration used for each experiment.

### 2.5. Leakage Ability of AMPs

**Figure 5:**
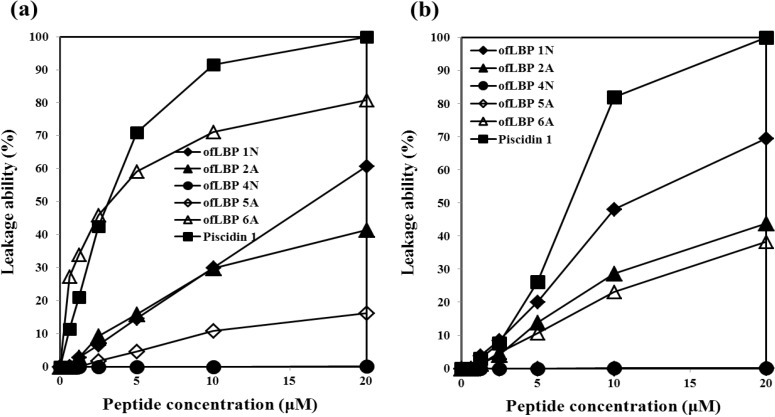
Release of encapsulated calcein from neutral liposomes including DOPC (**a**) and acidic liposomes including DOPC/DOPG (3:1) (**b**) measured for 3 min after the addition of peptides (0.63–20 μM).

To determine whether certain bacterial membrane compositions were preferentially targeted by ofLBP analogs, membrane perturbation was examined by measuring calcein release from artificial acidic liposomes including DOPC/DOPG (3:1) and artificial neutral liposomes including DOPC (Figure 5). ofLBP analogs showed significant leakage abilities compared to piscidin 1; perturbed neutral liposomes displayed higher leakage than acidic liposomes. In contrast, piscidin 1 caused complete leakage of entrapped calcein from both types of liposomes at a concentration of 20 μM. In the presence of neutral liposomes, ofLBP analogs (excluding ofLBP 4N and 5A) showed significant membrane perturbation abilities, although it was weaker than piscidin 1. By comparing the membrane perturbation abilities among ofLBP analogs, ofLBP 6A and 1N exhibited stronger leakage ability than ofLBP 2A and 5A. In the presence of acidic liposomes, ofLBP analogs (excluding ofLBP 4N and 5A) also showed significant membrane perturbation abilities, although the abilities were weaker than piscidin 1. By comparing the membrane perturbation abilities among ofLBP analogs, ofLBP 1N showed stronger leakage ability than ofLBP 2A and 6A. Membrane perturbation abilities of ofLBP analogs corresponded to the membrane permeabilization assay (Figure 4). Among the analogs, only ofLBP 6A demonstrated significant killing ability, which may have been due to the different peptide concentrations used for each assay: 26–40 μg/mL or 3–9 μg/mL was used for leakage and membrane permeabilization assays or killing assays, respectively. These results suggest that ofLBP analogs interact with the bacterial membrane, but whether they act directly on the membrane remains unclear.

### 2.6. Effect of Temperature on Antimicrobial Activity

**Figure 6:**
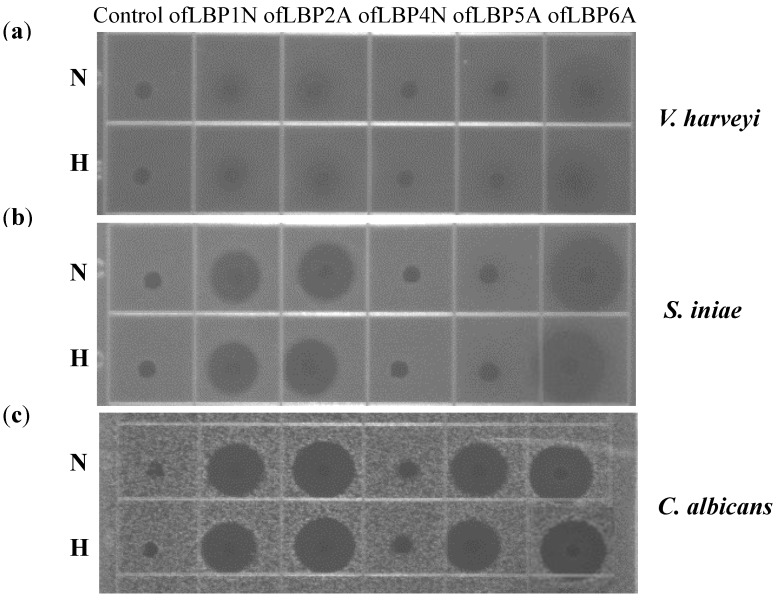
Thermal stability of ofLBP analog peptides against *Vibrio harveyi* (**a**); *Streptococcus iniae* (**b**); and *Candida albicans* (**c**). The upper and lower panels show the radial diffusion assay results of non-heated peptides (N) and after heating for 10 min at 100 °C (H).

To examine thermal stability, ofLBP analogs were incubated at 100 °C for 10 min. After heat treatment, the peptides were cooled and used for URDA against two bacteria including *V. harveyi* and *S. iniae*, as well as a yeast, *C. albicans*. Antimicrobial activity of ofLBP analogs was not significantly affected by heat treatment (Figure 6). When examining the antimicrobial activity of ofLBP analogs against *V. harveyi*, only ofLBP 6A showed significant activity. Antimicrobial activity of ofLBP analogs against *S. iniae* (ofLBP 6A, 2A, and 1N) showed significant activity. Antimicrobial activity of ofLBP analogs against *C. albicans* (ofLBP 6A, 2A, 1N, and 5A) also displayed significant activity. However, ofLBP 4N did not show any antimicrobial activity against the tested strains regardless of thermal treatment. The thermal stability investigations indicated that ofLBP analogs are heat-stable.

### 2.7. DNA-Binding Assay

**Figure 7:**
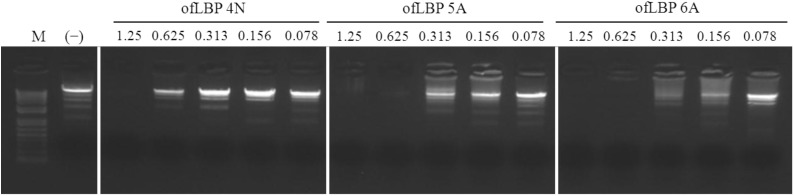
Gel retardation analysis or the binding of ofLBP analog peptides to DNA. Binding of peptides to DNA was assessed by measuring the retardation of commercial molecular weight marker λ-*Hind*III-digested DNA (50 ng) migration through an agarose gel. The peptide concentration is indicated above each lane and represents 1.25, 0.625, 0.313, 0.156, and 0.078 μg. Control (−) was without peptide.

To investigate the binding ability of ofLBP analogs to DNA, an electrophoretic mobility shift assay (EMSA) was performed with three ofLBP analogs: ofLBP 4N, 5A, and 6A. The electrophoretic mobility of the DNA was almost completely inhibited by ofLBP 6A or ofLBP 5A at 0.625 μg and ofLBP 4N at 1.25 μg, but was slightly inhibited by ofLBP 4N at 0.625 μg compared to non-complexed DNA (Figure 7). The Gln of LBP4N was substituted with Trp to create LBP5A, and LBP4N was modified to include a Met at the C-terminus to create LBP6A. No change occurred in net charge (+4), but the Boman Index, hydrophobic ratio, and hydrophobicity changed in ofLBP5A and 6A (Table 1), suggesting that the DNA-binding ability varied in proportion to the above three index values rather than the net charge [23].

### 2.8. DNA polymerase Inhibition Assay

**Figure 8:**
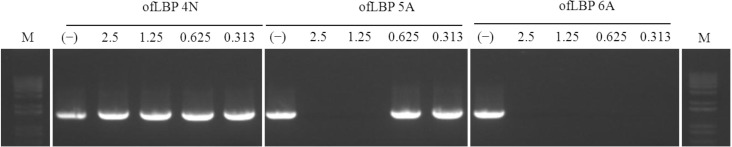
DNA polymerase inhibition assay of ofLBP analog peptides. The effect of peptides on DNA polymerase was tested by PCR for amplifying *Escherichia coli* 16S rDNA. The peptide concentration is indicated above each lane and represents 2.5, 1.25, 0.625, and 0.313 μg. Control (−) was without peptide.

To determine whether the peptides inhibit DNA polymerase activity, DNA polymerase inhibition assays were performed using three ofLBP analogs: ofLBP 4N, 5A, and 6A (Figure 8). ofLBP 5A and ofLBP 6A showed strong DNA polymerase inhibitory ability but ofLBP 4N displayed no DNA polymerase inhibitory ability. ofLBP 6A exhibited complete inhibitory activity at all tested concentrations (0.313–2.5 μg), but ofLBP 5A showed less inhibitory activity at 1.25 μg compared to ofLBP 6A, suggesting that ofLBP 6A has stronger binding ability to DNA polymerase than DNA alone, while ofLBP 5A may have stronger binding ability to DNA than DNA polymerase. However, ofLBP 4N may have a weak binding ability to DNA alone and the DNA polymerase.

## 3. Experimental Section

### 3.1. Peptide Synthesis and Purification

The olive flounder LBP/BPI-derived peptide analogs (ofLBP1N, ofLBP2A, ofLBP4N, ofLBP5A, and ofLBP6A) were commercially synthesized by Peptron Inc. (Daejeon, Korea) at a purity grade of >95%. Briefly, the peptide was synthesized using Fmoc solid phase peptide synthesis (SPPS) with ASP48S (Peptron Inc., Daejeon, Korea) and purified using reverse phase high-performance liquid chromatography (HPLC) with a Vydac Everest C18 column (250 mm × 22 mm, 10 μm; Grace, Deerfield, IL, USA). Elution was performed with a water–acetonitrile linear gradient (3%–40% (v/v) of acetonitrile) containing 0.1% (v/v) trifluoroacetic acid. Molecular weights of the purified peptides were confirmed using liquid chromatography/mass spectrometry (LC/MS; HP1100 series; Agilent, Santa Clara, CA, USA). All synthetic peptides were dissolved in 0.01% acetic acid to obtain stock solutions of 1000 µg/mL.

### 3.2. Ultrasensitive Radial Diffusion Assay for Antimicrobial Potency

The antimicrobial activity of the purified peptide was assessed as described previously [24]. We tested the synthetic peptides against several Gram-positive bacteria including two *Bacillus subtilis* strains, *Micrococcus luteus*, *Staphylococcus aureus*, and *Streptococcus iniae*, Gram-negative bacteria including three *Escherichia coli* strains, *Aeromonas hydrophila*, *Edwardsiella tarda*, *Pseudomonas aeruginosa*, *Vibrio parahemolyticus*, and *Salmonella enteric*, as well as the yeast *Candida albicans*. All strains were grown overnight for 18 h in trypticase soy broth (TSB) or sabouraud dextrose broth (SDB) at the appropriate temperature (25 °C for *A. hydrophila*, *E. tarda*, *P. aeruginosa*, *S. iniae*, and *V. parahemolyticus* and 37 °C for the other strains). After an overnight incubation, the bacterial and *C. albicans* suspensions were diluted to a McFarland turbidity standard of 0.5 (Vitek Colorimeter #52-1210; Hach, Loveland, CO, USA) corresponding to ~10^8^ CFU/mL for bacteria and ~10^6^ CFU/mL for *C. albicans*. One-half milliliter of diluted bacterial or *C. albicans* suspension was added to 9.5 mL of underlay gel containing 5 × 10^6^ CFU/mL or 5 × 10^4^ CFU/mL in 10 mM phosphate buffer (PB; pH 6.6) with 0.03% TSB or 0.03% SDB and 1% Type I (low EEO) agarose. The purified peptide was serially diluted twofold in 5 μL of acidified water (0.01% HAc), and each dilution was added to 2.5-mm-diameter wells made in the 1-mm-thick underlay gels. After incubation for 3 h at either 25 °C (for *A. hydrophila*, *E. tarda*, *P. aeruginosa*, *S. iniae*, and *V. parahemolyticus*) or 37 °C (the other strains), the bacterial or yeast suspension was overlaid with 10 mL of double-strength overlay gel containing 6% TSB or 6% SDB with 10 mM PB (pH 6.6) in 1% agarose. Plates were incubated for an additional 18–24 h, after which the clearing zone diameters were measured. After subtracting the diameter of the well, the clearing zone diameter was expressed in units (0.1 mm = 1 U).

### 3.3. Minimal Effective Concentration of the ofLBP-Derived Analogs

All tested bacteria and yeast were prepared as described above. The minimal effective concentration (MEC, μg/mL) of the synthetic peptides was calculated as the *x*-intercept of a plot of units against the log_10_ of the peptide concentration [25]. Piscidin 1, an α-helical AMP isolated from hybrid striped bass (*Morone saxatilis* × *Morone chrysops*) kindly provided by E.J. Noga (North Carolina State University, Raleigh, NC, USA), was used as a positive control [26]. The antimicrobial assay was performed in triplicate and the results were averaged.

### 3.4. Hemolytic Activity Assay

The hemolytic activity of the peptides was determined using human red blood cells (RBCs, blood type O) [22]. The RBCs were collected from citric acid-treated blood by centrifugation at 3000× *g* for 5 min and washed three times with 10 mM PB (pH 7.4) containing 150 mM NaCl to remove the plasma and buffy coat. A suspension of 3% hematocrit in buffer with or without peptides was incubated for 60 min at 37 °C. Hemolysis was expressed as the hemoglobin content obtained from the absorbance of the supernatant at 405 nm after centrifugation at 3000× *g* for 5 min. A 100% hemolysis was determined based on hemoglobin release after the addition of 0.1% Triton X-100. The hemolysis percentage of the peptides was calculated using the following formula:

% Hemolysis = [(Abs_405nm_ in the peptide solution − Abs_405nm_ in buffer)/(Abs_405nm_ in 0.1% Triton X-100 − Abs_405nm_ in buffer)] × 100

Hemolytic assays were performed in triplicate and the results were averaged.

### 3.5. Killing Kinetic Assay

To determine the killing activities of ofLBP-derived analogs, kinetic studies were performed using *B. subtilis* KCTC1021 and *E. coli* ML35p at 1× MEC and 5× MEC concentrations. Overnight culture of *B. subtilis* KCTC1021 and *E. coli* ML35p were diluted in TBS to a cell density of 10^6^ CFU/mL and 1 mL of each suspension was exposed to each ofLBP-derived analog and incubated at 37 °C overnight. The resulting colonies were counted. Percent killing was calculated as the proportion of live bacteria at a given time point following the addition of peptide compared to the number of bacteria present prior to the addition of peptide. Control cultures of each bacterium were incubated without any peptide and assayed at time points corresponding to the test culture assays to ensure no spontaneous loss of viability. Killing kinetic assays were also performed in triplicate, and the results were averaged.

### 3.6. Membrane Permeabilization

The outer membrane-permeabilizing activity of peptides was determined using the 1-*N*-phenylnapthylamine (NPN) uptake assay as described by Koo *et al.* [21]. Briefly, an overnight culture of *E. coli* ML35p was transferred to fresh TSB medium and grown to A_600_ of 0.5–0.6. Cells were harvested, washed, and resuspended in the same volume of buffer (5 mM HEPES, pH 7.2, 5 mM KCN, and 5 mM glucose). Next, 20 μL of 0.5 mM NPN was added and mixed with 1 mL of cells, and peptide samples (40 μg/mL) were added after 3 min. The increase in fluorescence due to partitioning of NPN into the outer membrane was measured 3 min after the addition of peptides using a spectrofluorometer (LS 50B Fluorescence Spectrometer; PerkinElmer, Waltham, MA, USA) at an excitation wavelength of 350 nm and an emission wavelength of 420 nm at 25 °C. The extent of cytoplasmic membrane permeabilization was determined by measuring β-galactosidase activity in *E. coli* ML35p using *O*-nitrophenyl-β-d-galactopyranoside (ONPG), a nonmembrane-permeative chromogenic substrate [27]. Mid-logarithmic phase *E. coli* cells were washed in 10 mM sodium phosphate buffer (NaPB), pH 7.4, and resuspended in the same buffer with 1.5 mM ONPG. The hydrolysis of ONPG to *O*-nitrophenol over time was monitored spectrophotometrically at 420 nm following the addition of peptide samples. Membrane permeabilization assays were performed in triplicate and the results were averaged. Piscidin 1 and 0.01% HAc were used as positive and negative controls, respectively.

### 3.7. Liposome Preparation

For leakage experiments, large unilamellar vesicles (LUVs) (DOPC or DOPC-DOPG, 3:1) with encapsulated calcein were prepared using the extrusion method [22]. The desired mixtures of phospholipids were dried in glass tubes under nitrogen and then lyophilized overnight to obtain lipid films. The dry lipid films were suspended in leakage buffer (20 mM TES buffer containing 150 mM NaCl, pH 7.4) and 70 mM calcein, and then vortexed occasionally to disperse the lipids. The suspension was freeze–thawed in liquid nitrogen for 10 cycles and extruded 10 times through 0.1-μm polycarbonate membrane filters in an Avanti mini extruder apparatus (Avanti Polar Lipids, Inc., Alabaster, AL, USA). After extrusion, unencapsulated calcein was removed from the LUVs with encapsulated calcein using gel filtration on an 18-cm Sepharose 4B column equilibrated with leakage buffer. Fractions containing LUVs were used for calcein release measurements. Leakage measurements were performed at 25 °C.

### 3.8. Leakage Assay

To examine the kinetics of membrane perturbation by peptides and to determine whether a preferential targeting of membranes with specific compositions occurred, we measured the abilities of the peptides and piscidin 1 (as a positive control) to induce leakage of a fluorescent dye (calcein) from acidic liposomes including DOPC/DOPG (3:1, w/w) (synthetic phospholipids containing saturated fatty acid chains) liposomes and from neutral liposomes including DOPC liposomes. Leakage of calcein from liposomes was determined as described by Park *et al.* [22]. A liposome suspension (25 µL; final concentration 95 µM) containing encapsulated calcein and 50 µL of an appropriately diluted peptide solution in TES buffer were added to 20 mM TES buffer (pH 7.4) to yield a final volume of 1.0 mL. The increase in the fluorescence of calcein when leaking out of liposomes was monitored at an emission wavelength of 520 nm and an excitation wavelength at 490 nm. The change in fluorescence intensity was measured for 3 min after peptide addition. Complete release of calcein was achieved by adding 10 µL of 10% (v/v) Triton X-100. The percentage of dye release was calculated as follows:

Dye leakage (%) = 100 × (F − F_0_)/(F_t_ − F_0_)

Where F is the fluorescence intensity caused by the peptide and F_0_ and F_t_ are those with buffer alone and with 10% Triton X-100, respectively.

### 3.9. Effect of Temperature on Antimicrobial Activity

To investigate the effect of temperature on ofLBP analogs, antimicrobial activity was tested using an ultrasensitive radial diffusion assay (URDA) against two bacteria including *V. harveyi* and *S. iniae*, as well as a yeast, *C. albicans*. To explore thermal stability, the ofLBP analogs were incubated at 100 °C for 10 min. After heat treatment, the peptides were cooled and used for URDA as described above.

### 3.10. DNA-Binding Assay

DNA binding assays were performed as described previously with minor modifications. This test assessed peptide-DNA binding by examining the inhibition of the rate of migration of DNA bands through agarose gels [28,29]. A commercial molecular weight marker, λ-*Hind*III-digested DNA (50 ng) (Roche, Basel, Switzerland), was mixed with varying amounts of peptide (0 μg, 0.156 μg, 0.313 μg, 0.625 μg, and 1.25 μg) in 0.01% acetic acid, incubated at room temperature for 5 min, and then electrophoresed in 1.0% agarose gels containing 0.5 μg/mL ethidium bromide (EtBr).

### 3.11. DNA Polymerase Inhibition Assay

The ofLBP analogs were dissolved in 0.01% acetic acid at a concentration of 1000 μg/mL. To assess PCR inhibition, various concentrations of the ofLBP analogs (2.5, 1.25, 0.625, and 0.313 μg/mL) were added to the respective reaction mixtures. *E. coli* genomic DNA (100 ng) was used as a template for PCR with the following primer pairs: 16S-F1, 5′-CTCCTACGGGAGGCAGCAG-3′ and 16S-R3, 5′-CCAGGGTATCTAATCCTG-3′. The predicted amplicon is ~1.5 kb in length. Each PCR comprised 30 cycles each of 30 s at 95 °C, 30 s at 55 °C, and 1 min at 72 °C. After PCR, all amplicons were electrophoresed in 1.0% agarose gels containing EtBr.

### 3.12. Structure Prediction

The secondary structure of the peptides was predicted using the GOR method [17]. The theoretical isoelectric point (p*I*) and net charge were estimated with ExPASy’s ProtParam server [30]. The helical wheel diagrams were produced using EMBOSS pepwheel (European Bioinformatics Institute, Cambridge, UK) [31]. The Boman Index values [18] were calculated according to the online Antimicrobial Peptide Database [32].

## 4. Conclusions

We designed five novel AMPs named ofLBP1N, ofLBP2A, ofLBP4N, ofLBP5A, and ofLBP6A derived from the *C*-terminus of the LBP/BPI precursor of olive flounder. To increase antimicrobial activity, the Gly of ofLBP2A was substituted with Lys to create ofLBP1N, the Gln of ofLBP5A was substituted with Trp to create ofLBP4N, a Met residue was added to the *N*-terminus of ofLBP6A, and the Gln was substituted with Trp to create ofLBP4N. The biological activities of these five analogs were evaluated against biomembranes or artificial membranes for the development of candidate therapeutic agents.

Antimicrobial and hemolytic results indicated that the helicity or amphipathicity is crucial for the interaction of ofLBP analogs with bacterial membrane rather than cationity and that they have low cytotoxicity. Studies on the interaction of analogs with membranes indicate that ofLBP analogs interact with the bacterial membrane directly or indirectly, and that the inhibition mode of ofLBP 6A is a bactericidal process rather than a bacteriostatic process. However, the other analogs act through bacteriostatic processes. Studies on the interaction of the analogs with intracellular molecules indicate that ofLBP analogs may bind to DNA itself or DNA polymerase. These results suggest that ofLBP analogs interact with membranes or intracellular molecules and then exert antimicrobial activity. Among the analogs, ofLBP6A is a selective and potent broad-spectrum antimicrobial peptide with low hemolytic activity and high heat stability. Additionally, we demonstrated that ofLBP6A interacts with DNA alone and DNA polymerase *in vitro*. This inhibitory potential may also be relevant *in vivo*. Therefore, the peptide (especially ofLBP6A) may represent a promising template for the development of a novel AMP.

To date, the LPS-binding activity of LBP and LPS neutralizing activity of BPI are fully expressed by those *N*-terminal regions. In this study, we found the *C*-terminus of LBP/BPI might be also contributing to antimicrobial activity. Further studies are needed to determine structural aspects and better understand the mechanisms relating to their antimicrobial activity.
